# Neutrophil-to-lymphocyte ratio and red blood cell distribution width to platelet ratio and their relationships with inflammatory and antioxidant status in dogs with different stages of heart failure due to myxomatous mitral valve disease

**DOI:** 10.1007/s11259-024-10431-y

**Published:** 2024-06-08

**Authors:** Meriç Kocaturk, Ahmet Saril, Abdullah Doğukan Oz, Camila Peres Rubio, Jose Joaquin Ceron, Zeki Yilmaz

**Affiliations:** 1 Department of Internal Medicine, Faculty of Veterinary Medicine, Bursa Uludag University, 16059 Bursa, Turkey; 2 Department of Animal Medicine and Surgery, Faculty of Veterinary Medicine, Regional Campus of International Excellence “Campus Mare Nostrum”, University of Murcia, 30100 Espinardo, Murcia Spain

**Keywords:** Complete blood cell indices, NLR, RDW/PLT, Inflammation, Heart failure, Dogs

## Abstract

We aimed to evaluate the red blood cell distribution width-to-platelet ratio (RDW/PLT) with other complete blood cell count (CBC) indices and their correlations with serum proinflammatory cytokines, acute phase proteins (APPs), and antioxidant biomarkers in dogs at different stages of heart failure (HF). A total of 29 dogs were divided into four groups according to the ACVIM Consensus Statement: stage-A (healthy/controls, *n* = 8), stage-B2 (*n* = 6), stage-C (*n* = 10), and stage-D (*n* = 5). Seventeen CBC indices were calculated and correlated with the measurements of inflammatory, APPs, and antioxidant biomarkers, as well as selected echocardiographic variables in all dogs. At stage-C, CBC indices were evaluated 14 days after the treatment. Statistically significant changes were observed only for RDW/PLT and neutrophil-to-lymphocyte ratio (NLR) between groups. NLR increased, but RDW/PLT deceased in dogs with HF, compared to controls (*P* < 0.05). There were no statistically differences between pre- and post-treatment CBC indices. There were significantly positive and negative correlations between the CBC indices, serum parameters and selected echocardiographic variables in dogs with HF(*P* < 0.05). ROC analysis showed the best sensitivity (57% and 68%) and specificity (100% and 57%) for NLR > 5.8 and RDW/PLT ≤ 0.057 for predicting the severity of HF, respectively. Results showed that NLR and RDW/PLT may have potential for monitoring severity of the disease and the effect of treatment in dogs with HF. Imbalances between indices of circulating blood cells can contribute to immunoinflammatory and antioxidant responses in pathogenesis of canine HF, which may provide us alternative targets to develop new diagnostic and therapeutic strategies in veterinary medicine.

**Supplementary Information:**

The online version contains supplementary material available at 10.1007/s11259-024-10431-y.

## Introduction

Chronic heart failure (CHF) is mostly related with genetic and environmental risk factors, but also has close relation with inflammation. Inflammatory cells and signaling pathways are the pathological routes of development and progression of canine CHF (Rubio et al. 2020). Besides evaluating the biomarkers of the inflammation such as cytokines (interleukins) and acute phase proteins (APPs; C-reactive protein [CRP], haptoglobin [Hp]), and ceruloplasmin [Cp]), ratio of the traditional complete blood cell (CBC) indices seems to give significant information on diagnosis and prognosis in patients with different diseases including cardiopulmonary disorders, such as myxomatous mitral valve disease (MMVD) in dogs (DeProspero et al. 2023; Jung and Kim 2023; Ku et al. 2023) and hypertrophic cardiomyopathy (HCM) in cats (Fries et al. 2022).

Among several CBC indices, the neutrophil-to-lymphocyte ratio (NLR), monocyte-to-lymphocyte ratio (MLR), and platelet-to-lymphocyte ratio (PLR) are accepted as important systemic inflammatory indicators. In human medicine, NLR was found to be associated with the severity of the disease in patients with dilated cardiomyopathy (DCM) (Avci et al. 2014) and in dogs with MMVD (DeProspero et al. 2023; Jung and Kim 2023; Ku et al. 2023). Previous human studies showed that NLR and PLR were not sufficient to establish a diagnosis of heart failure, but NLR could be used to predict mortality during the follow-up of patients with CHF (Durmus et al. 2015). In veterinary medicine, the changes in peripheral mononuclear cells such as neutrophilia, lymphopenia, leukocytosis and/or a regenerative left shift were reported in dogs with DCM (Vatnikov et al. 2019). Additionally, increased serum level of CRP and circulating WBC count were thought to be associated with inflammation in response to advanced cardiomyopathy in dogs (Petric et al. 2018; Svete et al. 2021). Increased red blood cell distribution width (RDW) in dogs with pulmonary hypertension was reported and tricuspidal regurgitant pressure gradient was found to be associated with RDW (Mazzotta et al. 2016).

In addition to cardiac damage biomarkers such as cardiac troponins and natriuretic peptides, CBC indices were investigated in order to evaluate the severity of heart failure and coronary artery diseases and to evaluate their prognostic performance by several previously published studies in human medicine (Angkananard et al. 2021; Avci et al. 2014; Durmus et al. 2015; Ozyilmaz et al. 2017) and veterinary medicine (DeProspero et al. 2023; Fries et al. 2022; Guglielmini et al. 2013, 2021; Jung and Kim 2023; Ku et al. 2023; Kumiega et al. 2020). The red blood cell distribution width-to-platelet ratio (RDW/PLT), a combination of two independent parameters (RBCs and PLTs), is accepted as a new index to reflect the severity of inflammation and is suggested as a diagnostic and prognostic parameter in humans and dogs with cardiovascular diseases (Celik et al. 2016). Although the value of some CBC indices in the dogs with different stages of heart failure due to MMVD were reported, the diagnostic and treatment monitoring performance of RDW/PLT, and possible correlations between CBC indices and serum immunoinflammatory and antioxidant biomarkers in those dogs have not been investigated yet. Thus, the aims of this study were to evaluate which CBC indices could be better to describe disease severity and to assess prognosis in dogs with MMVD at different severity, according to the American College of Veterinary Internal Medicine (ACVIM) consensus statements; and to investigate their potential interactions with serum proinflammatory cytokines, APPs, and antioxidant biomarkers, as well as monitoring the change in CBC indices of dogs after the treatment.

## Materials and methods

### Ethical approval

 The study was approved by the Animal Experiments Local Ethics Committee (protocol code and date of approval: 2018-05/02) of Uludag University (HADYEK). Informed consent was obtained and signed by all patient owners involved in the study.

### Study material

This study was performed between July 2018 and January 2020 at Bursa Uludag University (BUU) Veterinary Teaching Hospital (Bursa-Turkey) and approved by the Animal Experiments Local Ethics Committee of the BUU (ID: 2018-05/02).

This study consisted of a total of 29 client-owned dogs of different breed, age, body weight, and both sexes, with different stages of HF due to MMVD. MMVD was suspected based on the clinically detectable heart murmur and cardiomegaly in thoracic radiography, and mitral valve degeneration was confirmed by echocardiographic examinations. The dogs were staged according to the ACVIM classification of HF (Keene et al. 2019; Atkins et al. 2009); stage A (healthy/controls, *n* = 8), stage B2 (*n* = 6), stage C (*n* = 10), and stage D (*n* = 5). Criteria used for classifying groups and some of the clinical data (physical, hematological and serum biochemistry examinations and cardiac imaging findings) were already detailed in our (Rubio et al. 2020; Saril et al. 2022) and other previously published papers (DeProspero et al. 2023; Jung and Kim 2023). Briefly, MMVD B2 was defined when a dog did not show clinical signs relating to cardiopulmonary disease, in conjunctive with structural remodeling of the heart meaning the presence of cardiomegaly radiographically, and echocardiographic evidence of left atrial and/or left ventricular dilatation. If a dog showed the clinical signs with confirmed cardiac remodeling, it was classified according to responsive (stage C) or unresponsive to treatment (refractory HF, stage D).

Two weeks after initiation of appropriate medical therapy, all dogs were re-examined, but only hematological data collected from stage C dogs were used to evaluate changes in CBC values post-treatment. To ensure that cardiorenal syndrome, electrolyte disturbances, and hemoconcentration due to forced diuresis with multiple diuretic medications were excluded, only stage C dogs (chronic HF) were selected for reevaluation for CBC. Dogs with HF in stage C received standard treatment with dosage regimens recommended in the ACVIM guidelines for canine heart failure (Keene et al. 2019; Atkins et al. 2009) as it was reported in our previous study (Saril et al. 2022).

### Measurements

 All dogs included in this study had detailed anamnesis, physical examination, and laboratory analysis as part of the routine diagnostic procedures. CBC analysis with 24 parameters of the dogs in all groups were performed in the animal hospital laboratory within 1 h after blood collection (HM5, Abaxis). In all dogs, routine serum biochemistry panel with 14 parameters (Comprehensive Diagnostic Profile Rotor, VetScan®, Abaxis), serum thyroxine and cholesterol levels (T4/Cholesterol Reagent Rotor, VetScan®, Abaxis) and serum cTnI (cTnI cartridge, I-Stat®, Abaxis) were measured, as described in our previous papers (Rubio et al. 2020; Saril et al. 2022).

Seventeen CBC indices were manually calculated; monocyte-to-lymphocyte (MLR), neutrophil-to-lymphocyte (NLR), platelet-to-lymphocyte (PLR), hematocrit-to-plateletcrit ratio (HCT/PCT), red blood cell-to-hemoglobin ratio (RBC/Hgb), plateletcrit-to-lymphocyte ratio (PCT/Lym), red blood cell distribution width-to-lymphocyte ratio (RDW/Lym), mean corpuscular volume-to-lymphocyte ratio (MCV/Lym), RDW-to-platelet distribution width ratio (RDW/PDW), RDW-to-platelet ratio (RDW/PLT), mean platelet volume-to-platelet ratio (MPV/PLT), RDW-to-mean platelet volume (RDW/MPV), mean platelet volume-to-RDW (MPV/RDW) and mean platelet volume-to-plateletcrit ratio (MPV/PCT) in all dogs.

Serum concentrations of 13 cytokines including interleukin-2 (IL-2), IL-6, IL-7, IL-8, IL-10, IL-15, IL-18, interferon gamma-induced protein 10 (IP-10), monocyte chemoattractant protein 1 (MCP-1), granulocyte-macrophage colony-stimulating factor (GM-CSF), keratinocyte-derived chemokine (KC)-like, tumour necrosis factor-alpha (TNF-α) and interferon-gamma (IFN-γ) were measured using Milliplex® MAP magnetic bead panel (CCYTO-90 K Millipore, Billerica, MA) with an automated analyzer (Luminex 200®, Luminex Corporation, Austin, TX, USA), according to the manufacturer’s instructions as performed in our previous papers (Rubio et al. 2020; Saril et al. 2022).

Serum levels of acute phase proteins (APPs) including C-reactive protein (CRP), haptoglobin (Hp), paraoxonase 1 (PON1), and butyrylcholinesterase (BChE) were measured using the Olympus AU600 (Olympus Diagnostica GmbH) analyzer with commercially available methods, and their details were already given in our previous study (Rubio et al. 2020).

Oxidative stress biomarkers which included Trolox equivalent antioxidant capacity (TEAC), cupric reducing antioxidant capacity (CUPRAC), ferric reductase antioxidant power (FRAB), and thiol concentrations were determined in serum by using previously validated assays, as performed in our previous papers (Rubio et al. 2016a, b, 2020; Saril et al. 2022).

For the assessment of cardiac anatomy and function, transthoracic echocardiographic examinations were performed, using different modalities (2-D, M-mode, and color, CW/PW, and tissue Doppler) and different echo windows (RPSAx, RPLAx, subcostal, and left apical 4-5ch views), as reported in our previous studies (Rubio et al. 2020; Saril et al. 2022). Cardiac rhythm analysis was evaluated electrocardiographically using bipolar extremity derivation (Rubio et al. 2020).

### Treatment

 Two weeks after initiation of appropriate medical therapy, all dogs were re-examined, but only hematological data collected from stage C dogs were used to evaluate changes in CBC values post-treatment. To ensure that cardiorenal syndrome, electrolyte disturbances, and hemoconcentration due to forced diuresis with multiple diuretic medications were excluded, only stage C dogs (chronic HF) were selected for reevaluation for CBC. Dogs with HF in stage C received standard treatment with dosage regimens recommended in the ACVIM guidelines for canine heart failure, including ACE inhibitors such as benazepril (0.25–0.5 mg/kg, q12-14 h, po; Cibadrex, Abdi Ibrahim Ilaç San Ltd, Istanbul - Turkey), positive inotropes (pimobendan, 0.25 mg/kg, q12 hr, po; Vetmedin, Boehringer Ingelheim, Istanbul - Turkey), diuretics (furosemide, 1–2 mg/kg, q12 hr, po; Lasix 40 mg Tablet, Sanofi Aventis Ilaç Ltd, Istanbul - Turkey), as well as sodium-restricted diets such as Royal Canine Cardiac Dry, and/or cardiac supplement including Q10, carnitine and taurine (CardioVet Tablet, VetExpert) (Keene et al. 2019; Atkins et al. 2009). Additionally, in this study, treatments recommended in the literature were used for dogs with stage B2 (pimobendan, same dose for stage C) and D group HF (drugs used for stage C plus additional diuretics – spironolactone and/or torosemide with or without antiarrhythmic medication) (Keene et al. 2019; Atkins et al. 2009) as it was reported in a previous study (Saril et al. 2022). Follow-up CBCs of the stage C dogs were evaluated after 2 weeks of treatment initiation. Treatment regiments and dietary data of the dogs with different stages (A, B2, C, and D) of heart failure were given in Supplementary Table 1.

### Exclusion criteria

 In this study, MMVD dogs with either pulmonary hypertension or comorbidities such as hypothyroidism and cancer were not included. Additionally, since some medication could have affected on hemato-biochemistry profile analyzed in this study, the dogs received any kind of medication (steroids, non-steroids, antibiotics, positive inotropes or diuretics, etc.) or vaccination at least for 10 days before being admitted to the clinic were excluded from the study, as reported in our previous studies (Rubio et al. 2020; Saril et al. 2022).

### Statistical analysis

 Statistical analysis was performed by routine descriptive statistical procedures and software (SigmaPlot® Statistical and Graphing Software, California, USA). The changes of the analytes between healthy dogs (Stage A) and dogs with different stages of HF were assessed by One-way Analysis of Variance (ANOVA). The Pearson correlation coefficient (*r*) was used for measuring a linear correlation between CBC indices and cytokines, APPs, and antioxidant levels. Receiver operating characteristic (ROC) curve analysis was performed, using MedCalc (statistical software, https://www.medcalc.org, free trial) for ascertaining the prediction of NLR and RDW/PLT to distinguish normal dogs from symptomatic dogs. All data were expressed as median and inter-quartile range: Q1-Q3. Values of *P* < 0.05 were considered significant.

## Results

### Clinical and laboratory findings

 All data from studied dogs including physical examinations, electrocardiographic and echocardiographic examinations, and laboratory analysis (CBC, serum biochemistry panel, cytokine panels and oxidative stress biomarkers) were already given in detail in our previous studies (Rubio et al. 2020; Saril et al. 2022). Nevertheless, comparison of some selected clinical, echocardiographic, and hematological data of the dogs among different HF groups were given in Tables 1 and 2.

**Table 1:** Comparison of some selected clinical, echocardiographic, and serum biochemical data of the dogs with different stages (A, B2, C, and D) of heart failure (Median and inter-quartile range: Q1-Q3)

Parameter	A *n* = 8	B2 *n* = 6	C *n* = 10	D *n* = 5	PT *n* = 10	*P* value
Age (years)	3,5 3,0–4,0	6,5 2,0–10,0	9,0 4,25 − 13,7	9,0 5,5–14,0	-	0,054
Sex (M/F)	2/6	4/4	6/5	4/4	6/5	-
BW (Kg)	21,0 17,0–32,5	17,5 8,0–31,0	8,0 6,3–14,1	30,0 12,1–55,0	9,8 7,9–14,1	0,183
HR (bpm)	110,0 101,0–120,0	128,0 119,0- 144,0	160 146,5–171,5	163 148,0–239,5	148 127–158	0,009
RR (rpm)	36 24–46	60 56–64	68 57–75	60 54–74	60 52–66	< 0,001
T (ºC)	38,3 38,1–38,5	38,1 38,0–38,3	38,3 38,0–38,3	38,2 37,9–38,5	38,2 37,9–38,3	0,773
SBP (mmHg)	132 122,5-138,7	128 123,7-136,7	150 141,5-161,0	150 139,0-157,5	ND	0,047
LA (cm)	2,3^a^ 1,9 − 2,5	2,2^a^ 1,4 − 3,3	3,3^b^ 2,0–4,4	4,4^c^ 3,0–6,4	ND	< 0,001
LA/Ao	1,1^a^ 1,04 − 1,16	1,7 ^a^ 1,6 − 1,8	1,9^b^ 1,8 − 2,7	2,4^b^ 1,9 − 2,7	ND	< 0,001
LVDdN (cm)	1,3^a^ 1,0–1,6	1,7^b^ 1,6 − 1,8	1,7^bc^ 1,6 − 1,9	2,0^c^ 1,9 − 2,5	ND	< 0,001
LV FS %	33,0 28,0–37,5	34,5 32,0–38,0	28,0 25,2–36,5	23,0 16,5–34,0	ND	0,428
LV EF %	59,0 53,0–67,5	64,0 62,0–69,0	54,0 51,2–67,7	45,0 33,7–66,7	ND	0,296
MV E/A	1,9^a^ 1,8 − 2,1	2,2^ab^ 1,0–2,4	2,2^ab^ 2,1–2,5	3,0^b^ 2,5 − 3,6	ND	< 0.05
MR Vmax (ms)	Absent	1,1^a^ 1,8 − 1,6	3,3^b^ 2,9 − 3,8	4,2 ± 1,1^b^ 3,1–5,5	ND	< 0.01
TAPSE (cm)	0,8 0,7 − 1,2	0,8 0,7 − 0,8	1,1 1,0–1,8	1,4 1,1–1,5	ND	0,145
MAPSE (cm)	1,0 0,7 − 1,5	0,5 0,5 − 0,6	1,2 1,1–1,7	1,0 1,1–1,8	ND	0,167
EPSS (cm)	0,3 0,2 − 0,4	0,4 0,2 − 0,5	0,3 0,3 − 0,5	1,4 0,8 − 1,5	ND	0,135
cTnI (ng/dL)	0,02^a^ 0,02 − 0,04	0,03^a^ 0,01 − 0,03	1,64^b^ 0,53 − 2,73	1,18^b, c^ 0,26 − 9,99	ND	0,094
CHOL (mg/dL)	230 165,3–325,7	230,5 208,2- 268,2	217 203–272	170 136,2–279,7	ND	0,573
T4 (ng/dL)	2,6 2,1–3,6	2,3 2,0–2,0	2,4 2,3–3,4	1,9 1,9 − 3,6	ND	0,317

*BW* body weight, *HR* heart rate, *RR* respiratory rate, *T* temperature, *SBP* systemic blood pressure, *LA* left atrium, *Ao* aorta, *LA/Ao* left atrium to aorta ratio, *LVDdN* Normalized left ventricular diastole diameter, *LV FS* left ventricular fractional shortening, *LV EF* left ventricular ejection fraction, *MV E/A* Mitral Valve early filling pressure (E) and atrial contraction (A), *MR Vmax* Mitral regurgitation maximal velocity, millisecond (ms), *TAPSE* tricuspidal annular plane systolic excursion, *MAPSE* mitral annular plane systolic excursion, *EPSS* end-point-to septal separation, *CHOL* cholesterol, *T4* thyroxine, *cTnI* cardiac troponin I, *PT* Post-treatment (vs. stage C), *ND* not determined

^a, b,c^*P* < 0.05 compared to group A

**Table 2:** Comparison of some selected haematological data of the dogs among groups (Median and inter-quartile range: Q1-Q3)

Parameter	A *n* = 8	B2 *n* = 6	C *n* = 10	D *n* = 5	PT *n* = 10	*P* value
WBC × 10^3^/uL	10,44^a^ 9,2–12,7	9,0^a^ 8,3–11,2	16,5^b^ 12,6–29,9	19,7^b^ 16,11–21,7	14,7 10,9–17,7	0,004
Neu × 10^3^/uL	7,09^a^ 6,5–8,7	6,3^a^ 5,8–8,9	13,4^b^ 9,6–15,1	16,8^b^ 13,7–18,5	12,4^a, b^ 8,3–14,2	0,003
Mon × 10^3^/uL	0,5 ^a^ 0,3 − 0,7	0,4^a^ 0,2 − 0,6	0,8^a^ 0,4 − 1,1	0,9^b^ 0,5 − 1,5	1,24^b^ 0,7 − 1,3	0,029
Lym × 10^3^/uL	2,52 1,4 − 3,2	1,8 1,5 − 2,7	2,1 1,7 − 3,0	1,8 1,5 − 2,1	2,1 1,4 − 3,5	0,662
RBC × 10^6^/uL	6,5 6,1–6,9	7,2 7,0–7,2	6,3 5,5–7,0	5,5 5,2–6,7	6,2 5,1–7,6	0,193
HCT %	43,8 39,8–48,9	48,8 44,3–50,9	43,6 37,7–50,1	37,6 32,7–46,5	42,6 36,7–49,2	0,078
PLT × 10^3^/uL	296^a^ 215–346	352^a^ 297–574	322 283–472	295 218–387	334 272–495	0,024

*WBC* white blood cell, *RBC* red blood cell, *Neu* neutrophile, *Mon* monocyte, *Lym* lymphocyte, *HCT* haematocrite, *PLT* platelet, *PT* Post-treatment (vs. stage C)

^a, b^*P* < 0,05 compared to group A

ECG results showed the presence of sinus rhythm or respiratory sinus arrhythmia in healthy dogs (stage A), and B2 dogs with MMVD, but in symptomatic dogs (stage C and D), either sinus tachycardia (4/15) or atrial fibrillation (3/15) were detected. Echocardiography revealed that systolic function was preserved based on the FS%, EF%, MAPSE and TAPSE values in MMVD dogs, but left ventricular diastolic (restrictive) dysfunction was observed in symptomatic dogs (stage C and D) with MMVD. The severity of mitral valve regurgitation in symptomatic dogs was found higher than that of asymptomatic dogs with MMVD (Table 1).

### CBC indices

**Fig. 1:**
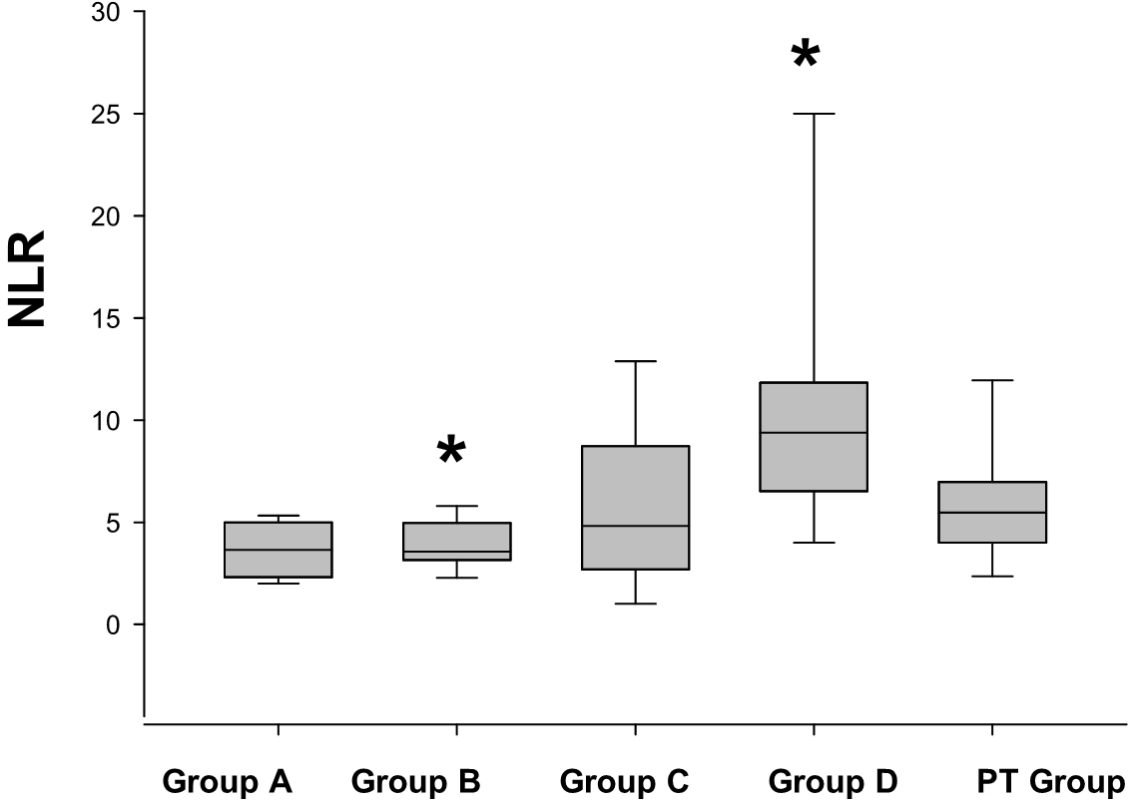
The neutrophil to lymohpcyte ration (NLR) of the dogs with HF according to ACVIM Classification compared to control group (Stage-A). PT: post treatment. Heart failure stages; A, B, C, and D (ACVIM classification). *P ≤ 0.05 compared to stage-A

**Fig. 2:**
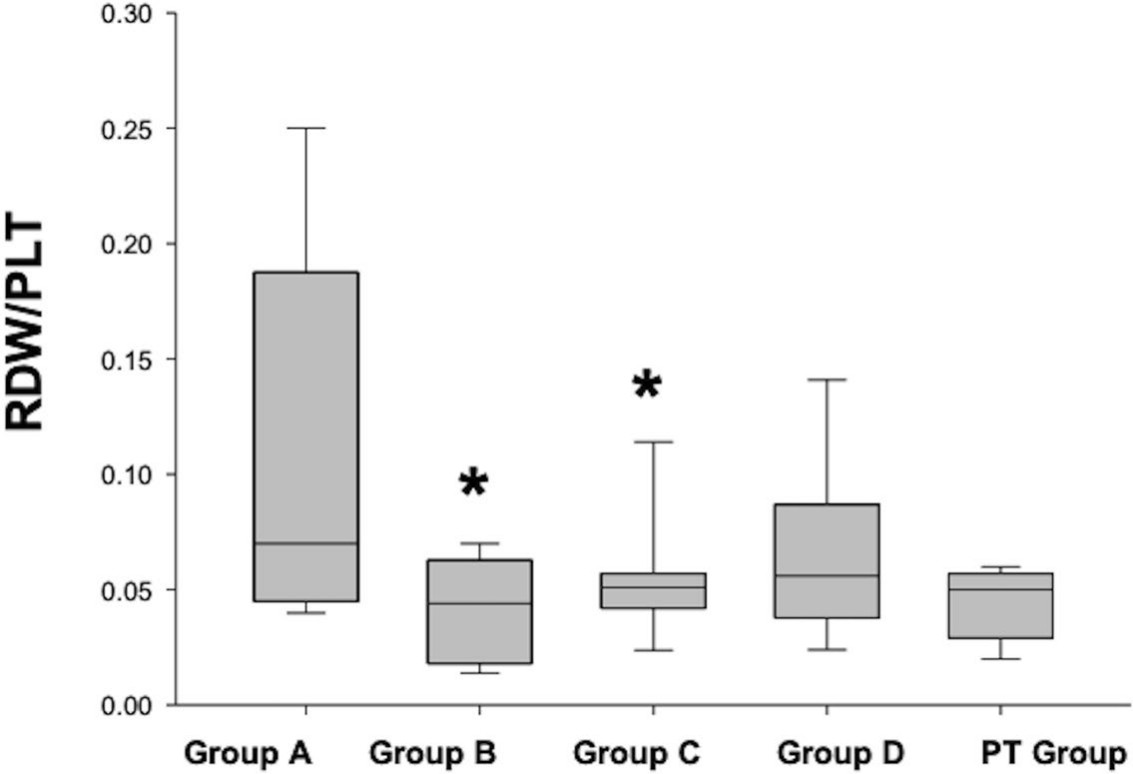
The reticulocyte definition width to platelet ratio **(**RDW/PLT) in dogs with HF according to ACVIM Classification compared to control group (Stage A). PT: post treatment. *P < 0.05 compared to stage-A

Only calculated CBC indices and post-treatment values which were not released in our previously published papers were given in this study (Table 3). Among all groups, NLR and RDW/PLT were found statistically significance difference in dogs with HF compared to controls. NLR was significantly increased in dogs of stage-B, -C and -D (*P* < 0.05 - <0.01) compared with stage-A (Fig. 1). RDW/PLT was decreased in dogs with stage- B and -C (*P* < 0.05) and there were no statistically differences in post-treatment group of dogs compared with stage-A dogs (Fig. 2).

**Table 3:** Complete blood cell indices of the dogs in different stage of heart failure according to ACVIM classification (Median and inter-quartile range: Q1-Q3)

Parameter	A *n* = 8	B2 *n* = 6	C *n* = 10	D *n* = 5	PT *n* = 10
MLR	0,21 0,12 − 0,26	0,22 0,1 − 0,38	0,32 0,19 − 0,34	0,37 0,24 − 1,21	0,46 0,21 − 0,62
NLR	3,6 ^a^ 2,3–5,0	3,5^a^ 3,2–4,9	4,8^b^ 2,7–8,7	9,4^c^ 6,5–11,8	5,5 4,0–6,9
PLR	118,4 81,6–198,4	295,0 113,8–699,6	157,6 109,2–280,0	178,6 112,0–306,5	127,0 96,0–307,7
RDW	16,9 16,6–17,7	16,9 16,3–21,4	17,1 16,4–17,8	16,2 15,6–19,9	16,6 15,8–17,4
HCT/PCT	142,7 116,8–167,2	105,6 69,5–168,4	130,7 102,1–247,4	121,4 102,1- 247,4	116,7 110,5–152,5
RBC/HGB	0,43 0,42 − 0,45	0,44 0,42 − 0,47	0,42 0,39 − 0,46	0,48 0,42 − 0,56	0,41 0,34 − 0,47
PCT/Lym	0,12 0,08 − 0,20	0,20 0,12 − 0,43	0,14 0,09 − 0,24	0,17 0,10 − 0,23	0,14 0,09 − 0,37
RDW/Lym	6,8 5,3–11,7	9,7 7,7–12,6	8,6 5,6–11,4	9,5 7,9–13,9	7,6 3,9–12,2
MCV/Lym	27,1 20,8–49,0	36,5 24,3–49,4	29,1 23,1–37,2	36,3 29,6–40,1	31,0 19,5–52.2
MPV/Lym	4,4 2,7 − 6,2	4,6 3,0–5,7	3,8 2,9 − 6,5	4,6 3,1–6,4	3,6 2,6–6,5
PDW/Lym	16,7 11,3–26,5	20,2 13,3–22,0	13,7 11,2–23,1	16,3 10,2–24,7	16,8 9,2–26,6
MCV/MPV	7,2 6,2–12,1	8,0 7,2–11,0	7,7 6,3–8,0	7,3 6,3–9,1	8,0 7,0–8,6
RDW/PDW	0,44 0,42 − 0,47	0,57 0,44 − 0,63	0,45 0,39 − 0,47	0,57 0,43 − 0,89	0,45 0,40 − 0,51
MPV/PLT	0,03 0,03 − 0,05	0,02 0,01 − 0,03	0,03 0,02 − 0,08	0,03 0,02 − 0,04	0,02 0,02 − 0,03
RDW/PLT	0,07^a^ 0,04 − 0,18	0,04^b^ 0,01 − 0,06	0,05^b^ 0,04 − 0,06	0,06^b^ 0,04 − 0,09	0,05 0,03 − 0,06
RDW/MPV	1,8 1,5 − 2,0	2,6 1,8 − 3,1	1,9 1,6 − 2,4	2,0 1,48 − 2,7	1,8 1,7 − 2,1
MPV/RDW	0,55 0,48 − 0,66	0,38 0,37 − 0,54	0,54 0,50 − 0,62	0,51 0,35 − 0,67	0,55 0,47 − 0,58
MPV/PCT	34,6 28,2–47,0	19,9 9,2–31,7	30,0 18,2–31,6	34,4 25,0–54,5	25,3 20,3–36,4

*MLR* monocyte to lymphocyte ratio, *NLR* neutrophil to lymphocyte ratio, *HCT/PCT* hemotoctrite to platelecrite ratio, *RBC/HGB* red blood cell to hemoglobin ratio, *PCT/Lym* platelecrite to lymphocyte ratio, *RDW/ Lym* red blood cell definition with to lymphocyte ratio, *MCV/ Lym* mean corpuscular volume to lymphocyte ratio, *MPV/ Lym* mean platelet volume to lymphocyte ratio, *PDW/Lym* platelet definition width to lymphocyte ratio, *MCV/MPV* mean corpuscular volume to mean platelet volume ratio, *RDW/PDW* red blood cell definition width to platelet definition width ratio, *MPV/PLT* mean platelet volume to platelet ratio, *RDW/PLT* red blood cell definition width to platelet ratio, *RDW/MPV* red blood cell definition width to mean platelet volume, *MPV/RDW* mean platelet volume to red blood cell definition width, *MPV/ RDW* mean platelet volume to reticulocyte definition width, *MPV/ PCT* mean platelet volume to platelecrite ratio

^a, b,c^*P* < 0.05 compared to group A (healthy controls)

### Correlations

 Since statistically significant changes were observed in only NLR and RDW/PLT among CBC indices between groups, these two parameters per se were used for the correlation study.

NLR of the dogs in stage-D was positively correlated to IL-10 (*r*: 0,999; *P*: 0,02) and IL18 (*r*: 0,999; *P*: 0,02) within the group. NLR of the dogs in stage-B was positively correlated to KC-Like (*r*: 0,919; *P*: 0,009) and negatively correlated to Hp (*r*: -0,825; *P*: 0,04) and IL-7 (*r*: 0,810; *P*: 0,05) within the group. NLR of the dogs in post-treatment group were positively correlated to LVDdN (*r*: 0,917; *P*: 0,01), CRP (*r*: 0,870; *P*: 0,02), BCHE (*r*: 0,981; *P*: 0,0005) and IP10 (*r*: 0,999; *P*: 0,02) and negatively to TEAC (*r*: 0,886; *P*: 0,01) and THIOL (*r*: 0,956; *P*: 0,0002) within the group.

RDW/PLT of the dogs in stage-C was positively correlated to IL-10 (*r*: 0,927; *P*: 0,007) within the group. RDW/PLT of the dogs in stage-D was positively correlated to CRP (*r*: 0,964; *P*: 0,001), Hp (*r*: 0,850; *P*: 0,03), FRAB (*r*: 0,964; *P*: 0,001), IL-2 (*r*: 0,906; *P*: 0,01), IL-7 (*r*: 0,919; *P*: 0,009), IL-15 (*r*: 0,977; *P*: 0,0007) and IL-18 (*r*: 0,972; *P*: 0,001) and negatively correlated to THIOL (*r*: -0,936; *P*: 0,005) and BCHE (*r*: -0,840; *P*: 0,03) within the group.

There were no correlations between NLR and/or RDW/PLT and serum IL-6, IL-8, PON-1 and CUPRAC concentrations.

### ROC curve analysis

**Fig. 3:**
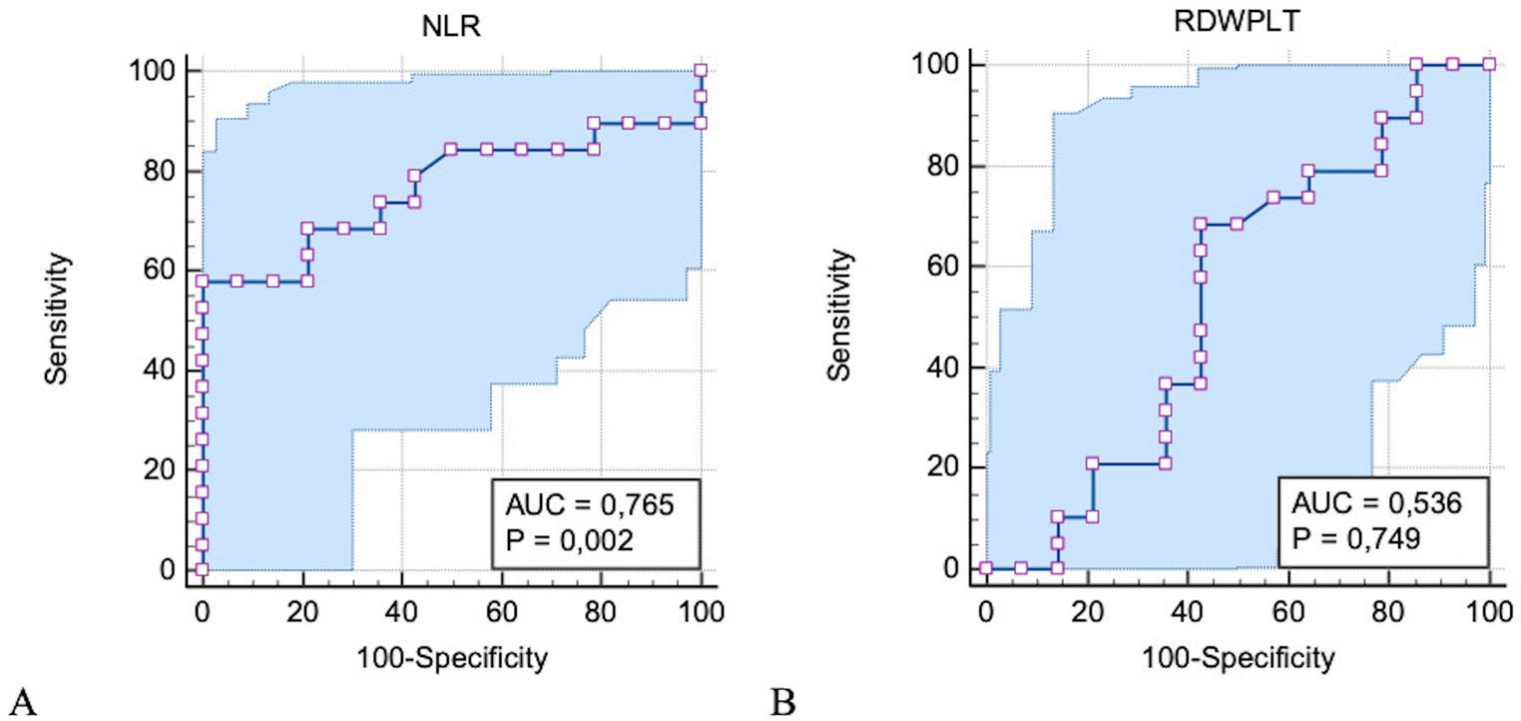
Receiver operating characteristic (ROC) curves for ascertaining the prediction of neutrophil-to-lymphocyte ratio (NLR) and red blood cell distribution width-to-platelet ratio (RDWPLT) to distinguish normal dogs from symptomatic dogs with MMVD (stage C and D). **A** ROC curve of NLR showed the area under the curve (AUC) value of 0.765 and the cut-point value of > 5.8. Therefore, dogs with NLR < 5.8 can be predicted as normal dogs with the sensitivity of 57% and specificity of 100%. **B** ROC curve of RDWPLT showed the AUC value of 0.536 and the cut-point value of ≤ 0,057. Therefore, dogs with RDWPLT ratio > > ratio ≤ 0,057 can be predicted as normal dogs with the sensitivity of 68% and specificity of 57%

 ROC curve of NLR showed the area under the curve (AUC) value of 0.765 and the cut-point value of > 5.8 with the sensitivity of 57% and specificity of 100% (Fig. 3A). ROC curve of RDW/PLT showed the AUC value of 0.536 and the cut-point value of ≤ 0,057 with the sensitivity of 68% and specificity of 57% (Fig. 3B).

## Discussion

 Herein, we focused on the CBC indices and their interactions with inflammatory and oxidative stress markers in dogs with different stages of HF. Because clinical, hemato-biochemical, and cardiological findings as well as inflammatory and oxidative stress status of this disease in dogs were discussed in detail in our (Saril et al. 2022; Rubio et al. 2020) and other previous studies (Atkins et al. 2009; DeProspero et al. 2023; Ku et al. 2023; Jung and Kim 2023). Our results show that among 17-calculated CBC indices, particularly NLR and RDW/PLT ratio may have a diagnostic and prognostic importance in dogs with MMVD. The relationships between CBC indices and serum levels of cytokines, APPs, and antioxidants indicated their potential roles in cellular, immunoinflammatory, and anti-oxidant mechanisms in development and progression of HF due to MMVD in dogs.

A CBC is a basic blood diagnostic test for in-house evaluation via auto-analyzers, and CBC-derived inflammation indexes (CBC indices) have an increasing popularity in diagnostic and prognostic evaluations in human and veterinary medicine from day to day. In this study, among CBC indices, we observed statistically significant changes only for NLR and RDW/PLT ratio between the dogs with different stages of HF. NLR is one of the most common parameters among them for diagnostic and prognostic evaluations in humans with cardiopulmonary disorders (Angkananard et al. 2021; Köse et al. 2020; Ozyilmaz et al. 2017) and sepsis (Ergenç et al. 2019; Spoto et al. 2021) and in dogs with MMVD (Ku et al. 2023; Kumiega et al. 2020), sepsis (Pierini et al. 2019, 2020), pancreatitis (Neumann 2021), meningoencephalitis (Park et al. 2022), chronic enteropathy (Becher et al. 2021) and inflammatory bowel disease (Benvenuti et al. 2020). In the present study, NLR was found significantly increased in dogs of stage-B, -C and -D compared to healthy controls (stage-A), which is parallel to the results from previously published studies in dogs (Jung and Kim 2023; Ku et al. 2023).

As with these pathological conditions, the NLR may also be increased in response to physiologic stress and/or external use of corticosteroids by increasing the number of circulating neutrophils, while decreasing the number of lymphocytes (Hodgson et al. 2018; Pierini et al. 2019; Ku et al. 2023). In the present study, observed increases in NLR may be related to the immuno-regulator roles of neutrophils in the different stages of MMVD. Because the pro-inflammatory roles of particularly neutrophils, and other blood cells (monocytes, lymphocytes, and platelets) in cardiovascular diseases are well known. That neutrophils secrete inflammatory mediators causes vascular wall degeneration and myocardial dysfunction, ultimately resulting in cardiac dysfunction (Jung and Kim 2023). Based on the accumulated evidence and our observations, it can be speculated that activated neutrophils play a cardinal role to orchestrate inflammation by modulating other inflammatory cells and releasing various cytokines (Jung and Kim 2023).

In the present study, RDW/PLT ratio decreased at statistically significant levels in asymptomatic (stage-B) and symptomatic dogs (stage-C); however, it did not show a statistical difference after the treatment in stage-C dogs. RDW/PLT ratio was suggested as an independent and novel marker for prognostic and mortality rate prediction in severe burn human patients (Qiu et al. 2017) and children with sepsis (Wang and Cai 2019). Also, RDW/PLT was found to be a strong predictor of the degree of fibrosis and cirrhosis in human patients with chronic hepatitis (Taefi et al. 2015). In one study, RDW per se was reported to be not associated with chronic degenerative valvular disease in dogs (Guglielmini et al. 2013), whereas in another, it was found to be an independent prognostic marker for negative outcomes in dogs with MMVD (Guglielmini et al. 2021). In addition, increased RDW was related with the severity of pulmonary hypertension in dogs (Mazzotta et al. 2016). It has been reported that increases of RDW was a result of cellular and cytokine mediated immune-inflammatory pathways of the HF (Hullin et al. 2019). Additionally, increase of RDW in HF was correlated with CRP and IL-6 in previously published human studies (Forhecz et al. 2009; Allen et al. 2010). In contrast to previous human and veterinary studies, in this study RDW alone was neither significant for predicting stages nor outcomes of the therapy in dogs with HF (Table 3). Since RDW/PLT has not been studied in previously published veterinary studies yet, in this study, when the RDW/PLT ratio was compared between HF groups, the decrease in group C and D compared to group A showed that this parameter would be used as supporting data in the prediction of the disease severity. Moreover, after the treatment of dogs in group C, it was observed that the RDW/PLT ratio was successfully normalized in the presence of compensated HF, showing that the RDW/PLT value may be useful in evaluating treatment success in dogs with HF.

In the present study, there were no statistically correlations between both CBC indices (NLR and RDW/PLT ratio) and echocardiographic geometric (LA and LV diameters and functional parameters (LV FS%). Parallel to our findings, in a recently published study, NLR was not found correlated with echocardiographic indices of systolic and diastolic functions (Ku et al. 2023). This observation shows that increased inflammation or stress could be associated with structural changes in the heart but not related to ventricular function in dogs with MMVD (Ku et al. 2023). Observed increases in the cardiac chambers in stage-C and -D dogs with HF could be explained by cardiac remodeling process and stress due to activation of compensatory neuro-humoral and immune-inflammatory mechanisms in response to end-stage HF (Rubio et al. 2020). In advance stage of dogs with HF, increased WBC, and neutrophil counts with increased serum level of CRP were found to be indicative of systemic inflammatory response (Rubio et al. 2020), however normal CRP concentration and normal WBC and neutrophil counts were also reported before (Petric et al. 2018). Thus, APPs, inflammatory cytokines and oxidative stress were reported to play important roles in the pathogenesis of canine HF (Saril et al. 2022).

In this study, correlations showed the presence of relationships between CBC indices, particularly NLR and RDW/PLT ratio, and serum levels of proinflammatory and anti-oxidant markers. Thus, NLR and RDW/PLT may be associated with end-stage HF, due to their potential roles in cellular immune and inflammatory mechanisms in development and progression of MMVD in dogs (Jung and Kim 2023). This observation could be supported by our previous findings including those inflammatory chemokines (KC-Like, and MCP-1, etc.) and APPs increased in severe stages of HF in dogs (Rubio et al. 2020). Similar to our results, significantly higher NLR was found in dogs with decompensated HF compared with compensated HF in dogs with MMVD (DeProspero et al. 2023; Ku et al. 2023; Jung and Kim 2023). These results would indicate that there is an on-going inflammation associated with HF which could be proven by CBC indices analysis, especially with NLR and RDW/PLT in dogs with chronic HF. Based on the ROC curve analysis, RDW/PLT seems to have higher sensitivity (68%) for a cut-off value of ≤ 0,057 compared to that (57%) of NLR of > 5.8, making RDW/PLT better to predict disease severity (asymptomatic vs. symptomatic) in dogs with HF.

There were some limitations in this study. One is that the number of samples was low in the groups and other is that the recovery and survival times of the dogs at different stages of HF could not be followed. In a recent study (Jung and Kim 2023), observed increases in some CBC indices (NLR, MLR, and PLR) were reported to be associated with the negative effects on survival in dogs with MMVD. Other studies from human and veterinary medicine showed that high RDW was associated with adverse prognosis in patients with HF (Xanthopoulos et al. 2022) and dogs with MMVD (Guglielmini et al. 2021). Thus, as with NLR and RDW, further studies are needed to understand whether RDW/PLT ratio has a potential for prognostic evaluation or to estimate survival time in dogs with MMVD. Although a previous study (Tangmahakul et al. 2023) reported that there were not statistically significant changes in RBC indexes (except MCH and MCHC) in dogs with MMVD complicated with pulmonary hypertension, whether the presence and severity of pulmonary hypertension and mitral regurgitation can affect on CBC indices such as NLR and RDW/PLT ratio needs to be clarify with further studies. The relationships between cardiac dysrhythmias such as atrial fibrillation and CBC indices should also be investigated in MMVD dogs, as reported in humans (Patel et al. 2020). Considering that it is important to include all these data in the prognostic assessment, large scale studies with higher number of dogs with HF needed to be studied.

## Conclusion

In conclusion, in this study, NLR and RDW/PLT ratio of the CBC parameters can be used in prognostic evaluation of HF in dogs and especially the NLR is a negative prognostic indicator and the RDW/PLT ratio can be useful in monitoring before and after treatment of the dogs with HF. This observation may give clinicians a different perspective in diagnostic and prognostic evaluations as well as in development of treatment strategies in patients with HF. Imbalances between indices of circulating blood cells can contribute to immunoinflammatory and antioxidant responses in the pathogenesis of HF. Further designed investigations are needed to determine the clinical usefulness of the NLR and RDW/PLT in dogs with HF due to MMVD.

### Electronic supplementary material

Below is the link to the electronic supplementary material.

Supplementary Material 1

## Data Availability

No datasets were generated or analysed during the current study.
