# Supplementary material for: Neutrophil-to-lymphocyte ratio and red blood cell distribution width to platelet ratio and their relationships with inflammatory and antioxidant status in dogs with different stages of heart failure due to myxomatous mitral valve disease
Source: Vet Res Commun. 2024 Jun 8;48(4):2477–87. doi: 10.1007/s11259-024-10431-y (PMC11315729; doi:10.1007/s11259-024-10431-y)
Supplement: Supplementary file 1 — Supplementary Material 1 [file 11259_2024_10431_MOESM1_ESM.docx]

**Supplementary table 1** Comparison of treatment regiments and dietary data of the dogs with different stages (A, B2, C, and D) of heart failure.

| GROUPS | A  n=8 | B2  n=6 | C  n=10 | D  n=5 | PT  n=10 |
| --- | --- | --- | --- | --- | --- |
| TREATMENTS | | | | | |
| Pimobendan | 0/8 | 6/6 | 10/10 | 5/5 | 10/10 |
| Enalapril/Ramipril | 0/8 | 0/6 | 10/10 | 5/5 | 10/10 |
| Furosemide | 0/8 | 0/6 | 10/10 | 2/5 | 10/10 |
| Hydrochlorothiazide | 0/8 | 0/6 | 0/10 | 5/5 | 3/10^b^ |
| Spironolactone | 0/8 | 0/6 | 0/10 | 5/5 | 3/10^b^ |
| Torasemide | 0/8 | 0/6 | 0/10 | 5/5 | 0/10 |
| Antiarrhythmic^a^ | 0/8 | 0/6 | 2/10 | 3/5^a^ | 2/10^b^ |
| DOSAGES | | | | | |
| Pimobendan | - | 0.25 mg/kg, q12 hr | 0.25-0,4 mg/kg, q12 hr | 0,4 mg/kg, q12 hr | 0.25-0,4 mg/kg, q12 hr |
| Enalapril/Ramipril | - | - | 0.25-0.5 mg/kg, q12-14 hr | 0.25-0.5 mg/kg, q12-14 hr | 0.25-0.5 mg/kg, q12-14 hr |
| Furosemide | - | - | 1-2 mg/kg, q12 hr | 4-6 mg/kg, q12 hr | 4-6 mg/kg, q12 hr |
| Hydrochlorothiazide | - | - | - | 2 mg/kg, q12 hr | 2 mg/kg, q12 hr |
| Spironolactone | - | - | - | 1-2 mg/kg, q24 hr | 1-2 mg/kg, q24 hr |
| Torasemide | - | - | - | Compensation of congestion: 0,1-0,6 mg/kg, q24 hr  For maintanence; 0,1 mg/kg, q24 hr | - |
| Antiarrhythmic^a^ | - | - | Digoxin and/or diltiazem^a^ | Digoxin and/or diltiazem^a^ | Digoxin and/or diltiazem^a^ |
| Cardiac diet* (CD) and/or supplements (CS)** | - | CD | CD and CS (< 15 Kg; 2 tablets in a day, >15 Kg; 4 tablets in a day) | CD and CS (< 15 Kg; 2 tablets in a day, >15 Kg; 4 tablets in a day) | CD and CS (< 15 Kg; 2 tablets in a day, >15 Kg; 4 tablets in a day) |

PT: post-treatment

^a^ Digoxin (0.005 – 0.008 mg/kg, q12 hr, PO) and/or diltiazem (0.5-1 mg/kg, q8-12hr, PO).

^b^ added according to requirement of the dogs based on the cardiac observations (radiography, ECG, and echocardiography) during re-examinations, two weeks later from initial treatments

*Commercial cardiac diet (Royal Canine Cardiac^R^), ** Cardiac supplement includes Q10, L-carnitine, Vit E, and Taurine (Cardiovet, Vet Expert^R^)
